# Quantitative characterization of the failure behavior of dangerous rocks based on the Frozen-Thawing Test

**DOI:** 10.1038/s41598-023-37703-y

**Published:** 2023-06-28

**Authors:** Ke Zhang, Yi Xu, Kai Zhang, Rui Bao, Wenchen Fan

**Affiliations:** 1 Faculty of Civil Engineering and Mechanics, Kunming University of Science and Technology, Kunming, 650500 Yunnan China; 2 Faculty of Electric Power Engineering, Kunming University of Science and Technology, Kunming, 650500 Yunnan China; 3 Kunming Prospecting Design Institute of China Nonferrous Metals Industry Co., Ltd, Kunming, 650051 Yunnan China; 4 School of Civil Engineering, Central South University of Forestry and Technology, Changsha, 410004 Hunan China

**Keywords:** Civil engineering, Structural materials, Theory and computation, Optical techniques

## Abstract

A deep knowledge of the failure mechanisms and early warning of dangerous rocks is an important issue in geological disaster prevention and reduction. This study focuses on the failure analysis of dangerous rocks from a laboratory scale, whose models are prepared by 3D printing (3DP) technology. The frozen–thawing test (FTT) is performed to reproduce the failure processes of toppling and falling types dangerous rocks. In addition, the digital image correlation (DIC) technique is applied to detect the deformation characteristics of dangerous rock models during the tests. The relative displacements along the structural plane and the displacement vectors on the dangerous rock surface are further extracted to quantitatively reveal the failure mechanism from a fine-view perspective. It is found that the toppling type dangerous rock is dominated by the rotational failure, while the falling type dangerous rock is dominated by tensile‒shear failure. Furthermore, a DIC-based early warning method is proposed for identifying the precursors of dangerous rock instability from a laboratory perspective. The results provide an important application and reference value for the study of dangerous rock prevention and reduction.

## Introduction

Rockfalls, as one of the frequent geological hazards along mountainous areas, pose a serious threat to lives, facilities, environments and transportation corridors^1,2^. The presence of dangerous rocks is the main source for rockfalls. These dangerous rocks can easily separate from structural planes of bedrock under the influence of gravity and triggering factors (such as earthquakes, rainfall and strong wind)^3^. The high positions and concealment of dangerous rocks lead to uncertain failure, and also, those rocks have high kinetic energy and high migration rates after instabilities^2,4^. Therefore, a deep knowledge of the mechanical characteristics and failure evolution of dangerous rocks is important to mitigate rockfall hazards.

A series of theoretical and numerical works have been carried out on the mechanical properties and failure mechanisms of dangerous rocks. Zhang et al.^5^ developed a computer program based on the displacement discontinuity method (DDM) to simulate the progressive failure of overhanging dangerous rocks under differential weathering. Wu et al.^6^ adopted the maximum shear stress method in combination with the Mohr‒Coulomb criterion to evaluate the stability of sliding overhanging rock. Huang et al.^7^ proposed an analytical method to determine the stress intensity factor (SIF) of overhanging dangerous rocks. Spreafico et al.^8^ used a simplified discrete fracture network (DFN) model coupled with the finite element Voronoi approach to reveal the secondary toppling mechanism of dangerous rocks. Bolla and Paronuzzi et al.^9^ performed 2D and 3D numerical simulations based on the limit equilibrium method to analyze the mechanical behavior of unstable rock slopes. Shi et al.^10^ evaluated the stability of dangerous rocks under different conditions by the block discrete-element method (DEM) combined with strength reduction. Wu et al.^11^ modeled the role of water/ice in crack development from a fracture mechanics viewpoint using the extended finite element method, and then explained the seasonality of dangerous rock collapse. Yin et al.^12,13^ presented a method to analyze the damage evolution process of thick-layer dangerous rock masses considering the reservoir water level change. However, these numerical simulation results are determined by the relative computational parameter values, so it is difficult to ensure their reliability. Meanwhile, the calculation of the theoretical analysis results is quite complicated. Laboratory testing, as an effective mean to realistically reproduce the deformation and failure process of dangerous rocks, has been rarely utilized due to the difficulties in model preparation.

In the past few years, 3D printing (3DP) technology has been applied for the preparation of model specimens due to its powerful capability in the high-precision manufacture of complex samples with special external shapes and internal structures. Based on X-ray computerized tomography (micro-CT) and 3DP technology, Zhu et al.^14^ prepared resin specimens whose characteristics are similar to those of natural volcanic rocks. Park et al.^15^ printed a geological physical model based on the Los Angeles Basin using stainless steel powder as the printing substrate. Que et al.^16^ combined the Voronoi diagram and 3DP technology to produce an irregular columnar jointed mold, which enabled the high-precision preparation of columnar jointed rock masses with different dip directions and angles. Song et al.^17^ proposed two methods for modeling joints that replicate natural rock morphology and lithology by combining 3D scanning, 3D printing, and 3D carving technologies. Wang et al.^18^ printed different sizes of rock masses with built-in major fracture and fracture networks by the 3DP technique. The existence of 3DP technology provides favorable conditions for studying the failure mechanisms of dangerous rocks from laboratory perspectives.

As technology continues to improve, scholars are gradually combining advanced measurement techniques to analyze the deformation and failure behavior of rock mass models from qualitative and quantitative perspectives. The digital image correlation (DIC) method, as a non-contact, non-destructive observation technique, has been rapidly developed in the field of rock mechanics testing^19–25^. However, DIC technology has yet to be used to their full potential in the study of the deformation and failure process of dangerous rocks.

In this study, the stereolithography apparatus 3DP technology is used to prepare the dangerous rock models with complex geometry. The frozen-thawing test method is adopted to reproduce the failure process of dangerous rocks. The progressive failure processes were tracked with the help of DIC technique. The failure mechanisms of dangerous rocks were studied by analyzing the displacement information extracted from the displacement fields. Specifically, the overall movement trend of the dangerous rock is obtained from the displacement vector, while the relative displacement along the joint plane is calculated to reflect the deformation of key parts from a more fine-view perspective. In addition, the anomalous precursors of relative displacement were identified. Finally, a new method for early warning of dangerous rocks destabilization was proposed. These laboratory findings are of some reference value for the prevention and reduction of rockfall hazards. Also, it promotes the intersection and integration of 3D printing and geological hazard research.

## Experimental method

### Engineering background and conceptual models of dangerous rocks

**Figure 1:**
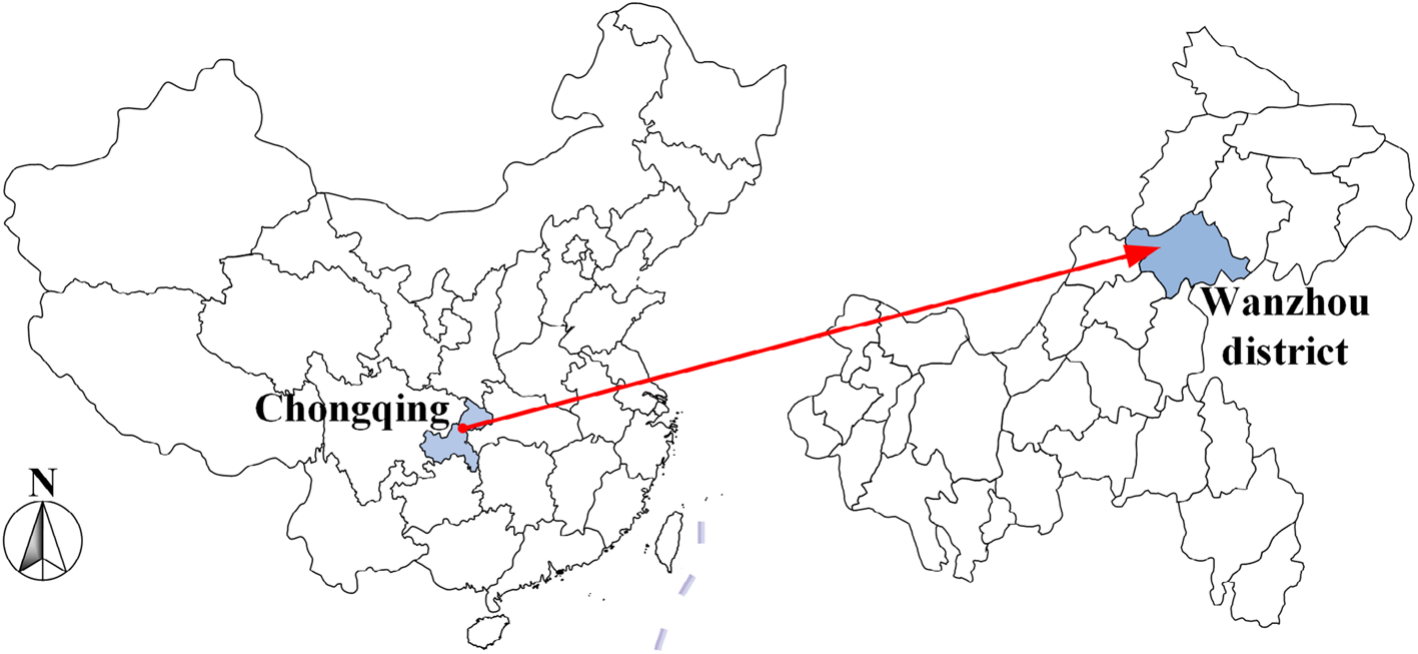
Location map of Taibaiyan dangerous rocks in Wanzhou, China.

The Taibaiyan dangerous rock is located in the Wanzhou district, Chongqing, China, as shown in Fig. 1, which is a typical rock fall-prone area. The rock formations of Taibaiyan are nearly horizontal under the influence of horizontal sedimentation. The main lithologies of the formations are Jurassic hard sandstone and soft mudstone. Soft mudstone has poor resistance to weathering and is prone to the development of rock cavities^26^. The development of rock cavities is a key factor in the destabilization of dangerous rocks in the Taibaiyan region^27^.

At present, 127 dangerous rock blocks are identified, with a total volume of approximately 81 186 m^3^, which are distributed in the middle section, the east section and the south slope of Taibaiyan. Among them, the south slope of Taibaiyan is developed, and 61 dangerous rock blocks are distributed, with a total volume of 24 562 m^3^. The presence of hazardous rock blocks can place the safety of facilities and personnel at the bottom at risk^5^. On May 19, 2004, a sudden failure of the W24 and W25 dangerous rock blocks in the south slope occurred, and a rock mass with a volume of approximately 2000 m^3^ destroyed the memorial, causing major losses. The toppling and falling failure modes are the most common in the Taibaiyan area.

**Figure 2:**
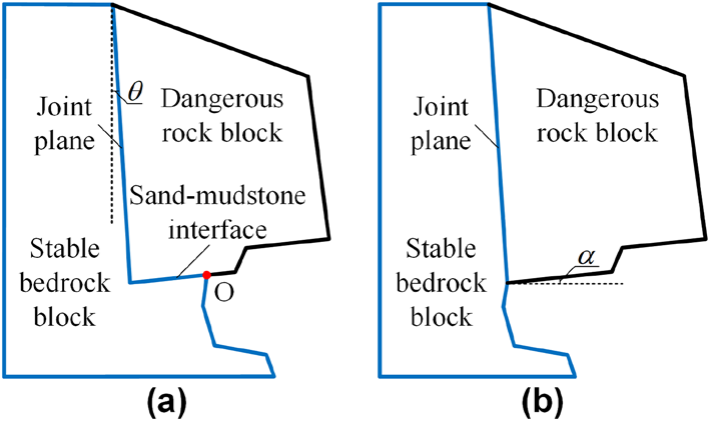
Conceptual models of dangerous rocks. (**a**) Toppling type dangerous rock. (**b**) Falling type dangerous rock.

Two conceptual models were established based on the two typical failure modes of dangerous rocks mentioned above, as shown in Fig. 2. Examples of first model are widely encountered in the Three Gorges Reservoir Region, People’s Republic of China^28^. The second model is obtained by expanding the undercutting depth on the basis of the first model. Although the cross section simplifies the prototype’s 3D geometry, it is considered to be representative and shows the critical features of the structure. The steep-tilted joint inclination is defined by *θ*, which are the angles of the joints relative to the vertical. The bedding joint inclination is defined by *α*, which are the angles of the joints relative to the horizontal. Each conceptual model is divided into two parts, including the dangerous rock block and stable bedrock block. For a dangerous rock model with a toppling failure mode, the gravity center of the dangerous rock block is located outside of the critical fulcrum (point O) at the stable bedrock block. For a dangerous rock model with a falling failure mode, the bottom of the dangerous rock block is completely free.

### Preparation of model specimens

**Figure 3:**
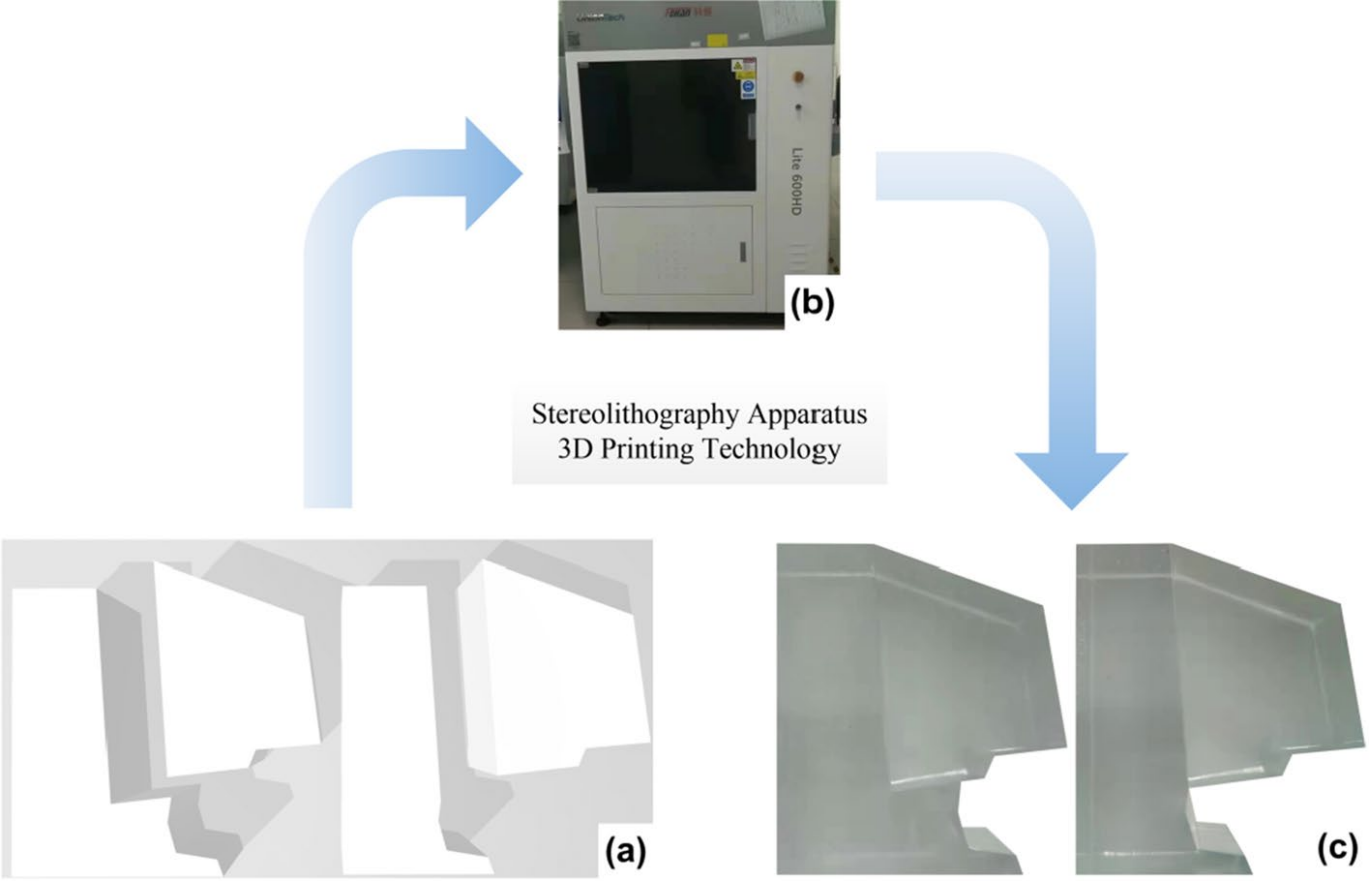
Schematic diagram of the 3DP process.

Currently, there are a few common 3D printing technologies in the field of rock mechanics as follows: fused deposition modeling, 3DP, selective laser sintering and stereolithography^18^. Compared to the other printing methods, the advantages of the stereolithography include smooth surface finishing, excellent optical clarity, high accuracy and excellent fine feature detail that require minimal finishing. Therefore, the stereolithography apparatus 3DP technology was used in this study to prepare the model specimens, where the printing equipment was an isLA660Lite Light-curing 3D printer, manufactured by Suzhou ZhongruiZhichuang 3D Technology Co., Ltd., China, as shown in Fig. 3b. The printer features a layer thickness of 0.05–0.15 mm and a maximum build volume of 108 L by using light curing technology. The printing material was photosensitive resin^29^. The printing process is described as follows:

*Step 1*: The conceptual models of the dangerous rocks were designed by AutoCAD software, and the model consisted of a dangerous rock block and a stable bedrock block, as shown in Fig. 2. The 3D dangerous rock digital model file was then converted to the STL format recognizable by the 3D printer, as shown in Fig. 3a.

*Step 2*: The obtained STL format file is loaded into *Magics* software and processed for layering. The print layer thickness was 0.1 mm, the optical scanning speed was 6.0 m/s, and the positioning accuracy of the lifting-lowering system was ± 0.01 mm.

*Step 3*: The digital model and printing parameters are uploaded to the isLA660Lite printer for printing. The formed specimens were removed after the completion of printing, and the resin on the specimen surfaces was washed with alcohol, as shown in Fig. 3c.

Random artificial speckle patterns were prepared on the target surface to meet the requirement of DIC calculation. First, a thin layer of white paint was sprayed evenly on the observation surface of the model specimen. Then, the black paint was randomly sprayed on the dried white paint to form black‒white speckle patterns.

### Experimental setup and procedure

The frozen-thawing test (FTT) method^30^ is used to reproduce the failure evolution of dangerous rocks from an indoor test perspective. The frozen ice layer is used to combine the dangerous rock block with the stable bedrock block as a whole model in this method. The gradual melting of the ice layer over time in a higher temperature environment leads to a decrease in bond strength, showing similarity in trend to the gradual deterioration of the joint strength over time characterized by the action of internal and external camp forces. Furthermore, the cracking and thawing of the ice layer will lead to the instability of dangerous rocks. Compared with the traditional dangerous rock failure simulation tests, the FTT method has clear advantages: (1) the whole process of gradual deterioration of joint strength is simulated effectively; (2) the physical parameters such as the weight of dangerous rock did not change during the test, and the test results are highly repeatable; (3) the experiment device and operation process are simple and the cost is low. Du et al.^31^ and Huo et al.^32^ reproduced the rock collapse process using FTT method.

**Figure 4:**
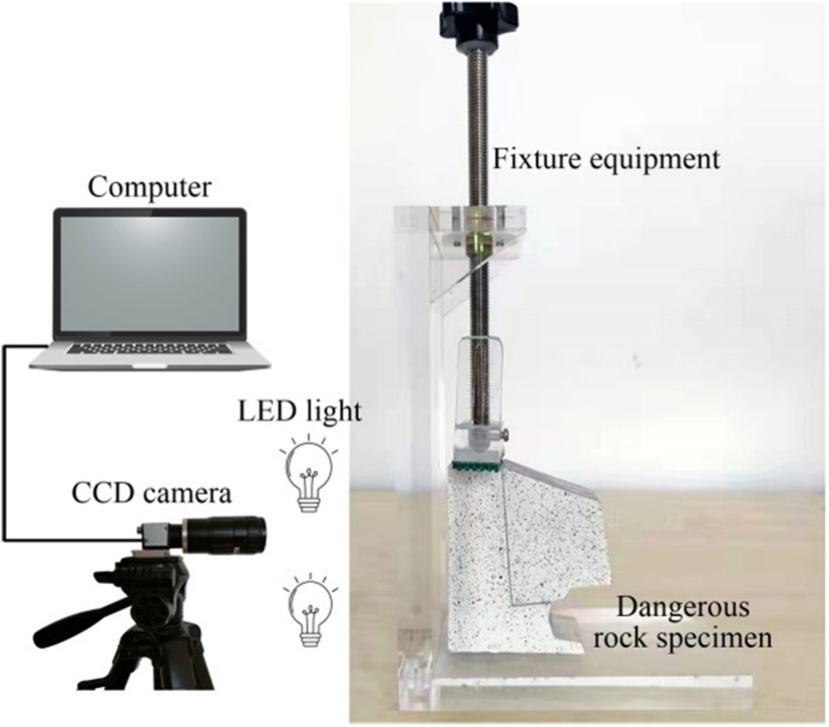
Test system.

Before the test, ice layers between the dangerous rock block and the stable bedrock block should be created first. Specifically, (1) the stable bedrock block and the dangerous rock block were submersed in pure water to reach saturated state; (2) after the water-saturated treatment, the stable bedrock block and the dangerous rock block were combined together and placed into the refrigerator, so that a cohesive ice layer is formed between the combined planes. It is worth noting that the refrigerator temperature is always set to − 15 °C to ensure consistent conditions for each freezing process. In addition, the mean value of ice thickness was measured to be 0.68 mm with a standard deviation of 0.02 mm through several attempts, indicating the high reproducibility of the freezing treatment. After the freezing processing was completed, the model was removed from the refrigerator and then placed on the fixture equipment (as shown in Fig. 4) for fixation. The fixture equipment ensures the stability of the stable bedrock block during the test. The speckle image acquisition system is built, including a computer, CCD camera, and LED lights, as shown in Fig. 4. The CCD camera is a 2592 × 1944 pixel industrial camera for the capture of speckle images. LED lights improve the clarity of the captured images. After the model specimen is fixed, the test is immediately started. The speckle image acquisition system is activated for speckle collection until the dangerous rock is destabilized. It should be noted that the room temperature is kept stable to ensure a consistent thawing rate of the ice layer. In this test, the room temperature is set to 15 °C.

## Results and analysis

### Deformation field evolution characteristics

**Figure 5:**
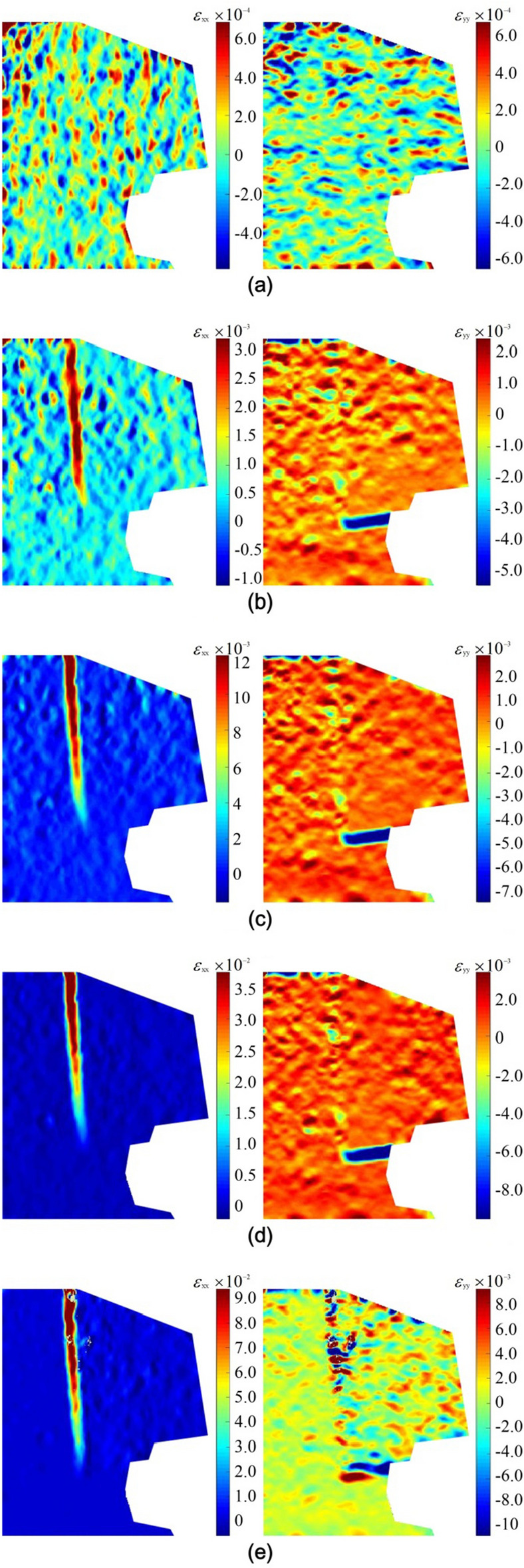
Strain field evolution of toppling type dangerous rock. (**a**) t = 200 s. (**b**) t = 725 s. (**c**) t = 840 s. (**d**) t = 950 s. (**e**) t = 995 s.

**Figure 6:**
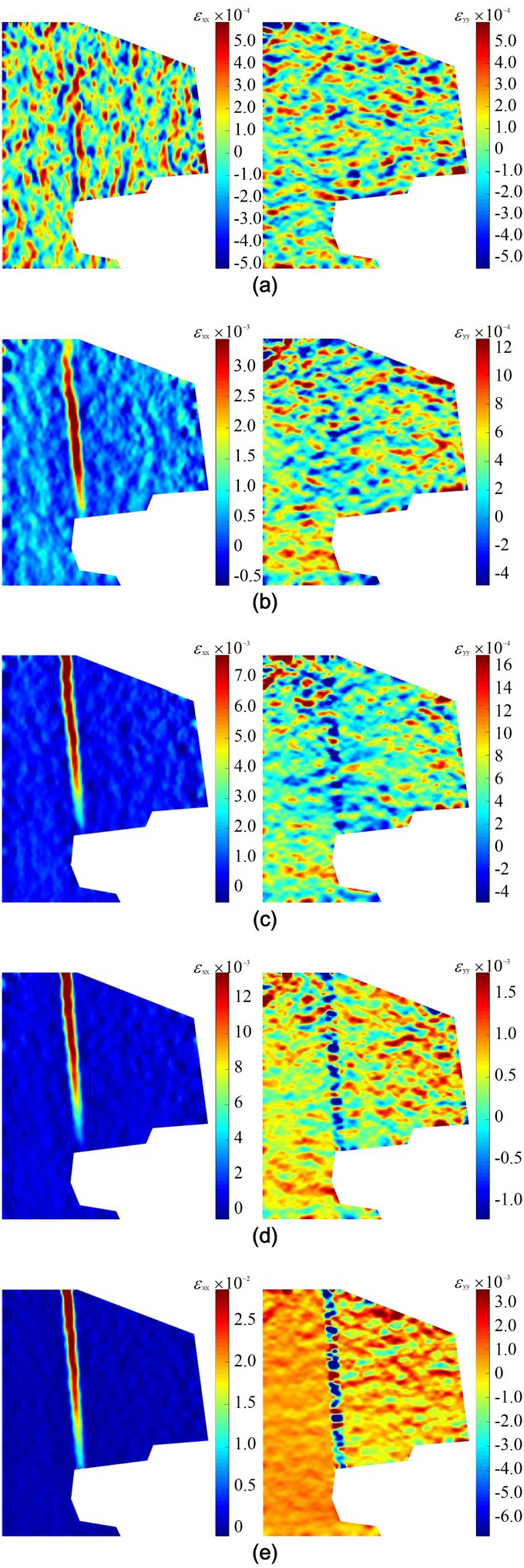
Strain field evolution of falling type dangerous rock. (**a**) t = 200 s. (**b**) t = 663 s. (**c**) t = 860 s. (**d**) t = 920 s. (**e**) t = 937 s.

The captured digital speckle images were imported into the Ncorr software for calculation to obtain the displacement field and strain field of the model specimen surfaces during the test. Ncorr software is an open-source 2D-DIC MATLAB program, that has well-documented capabilities and recognized solution practices. In this study, the subset size of 30 and the subset spacing of 2 are selected as Ncorr parameters. The image at the beginning of the test is used as a reference image for recreating the deformation field on subsequent images. The calculated horizontal strain fields (*ɛ*_xx_) and vertical strain fields (*ɛ*_yy_) on the model specimen surfaces at five typical moments are shown in Figs. 5 and 6. Two different failure modes of dangerous rock were reproduced in the test: the toppling failure mode and falling failure mode. The DIC measurements capture the full-field deformation of dangerous rock blocks, the joint plane (or sand-mudstone interface), and the stable bedrock block from the outside. Some quantitative and novel insights into the deformation and failure mechanisms of dangerous rocks can be obtained.

According to the strain field evolution shown in Figs. 5 and 6, the failure process of the toppling type and falling type dangerous rock can be divided into three stages:

1. Stable stage: As shown in Figs. 5a and 6a, the horizontal and vertical strain fields on the model surface are almost uniformly distributed, and there is no obvious strain concentration phenomenon. The strain values are very small. In this stage, the dangerous rock block is kept stable by the acting force provided by the ice layer at the joint plane (or sand-mudstone interface).
2. Weakening stage: As shown in Figs. 5b–d and 6b–d, the deformation of the joint plane (or sand-mudstone interface) is increasing continuously with the melting of the ice layer, i.e. the joint characteristics is continuously deteriorating, causing the separation trend between the dangerous rock block and stable bedrock block. In the horizontal strain fields of two dangerous rock models, strain localization phenomena along the joint plane are both observed. It is worth noting that strain localization exhibits different development paths. For toppling type dangerous rock, the strain concentration is initiated at the upper part of the joint plane. The strain localization zone gradually extends downward as the time increases. For falling type dangerous rock, the strain localization zone develops from the middle of the joint plane toward the two ends. The strain localization phenomenon along the sand-mudstone interface is also observed in the vertical strain fields of toppling type dangerous rock. In this stage, the strain values of dangerous rocks show a gradual increase, indicating that the stability of the dangerous rock decreases.
3. Destabilization failure: As shown in Figs. 5e and 6e, the ice layer strength at the joint plane is further weakened, resulting in the adhesive force generated by the ice layer not being sufficient to keep the dangerous rock block stable, and destabilization occurs. The horizontal and vertical strain values of the two dangerous rock models show growth. In particular, the localization phenomenon in the vertical strain field of toppling type dangerous rock shows obvious changes. Two different states of strain localization phenomenon are observed at the sand-mudstone interface, while the strain localization phenomenon at the joint plane is deflected, which indicates that the dangerous rock block of the toppling type dangerous rock is rotated.

From the evolution characteristics of the above strain field, it can be seen that the strain localization phenomenon can better reflect the deformation and failure state of the dangerous rock. In addition, the stabilization duration of the toppling type dangerous rock block is longer than that of the falling type dangerous rock block, because the bottom of the former is supported by stable bedrock.

### Evolution characteristic of relative displacement

**Figure 7:**
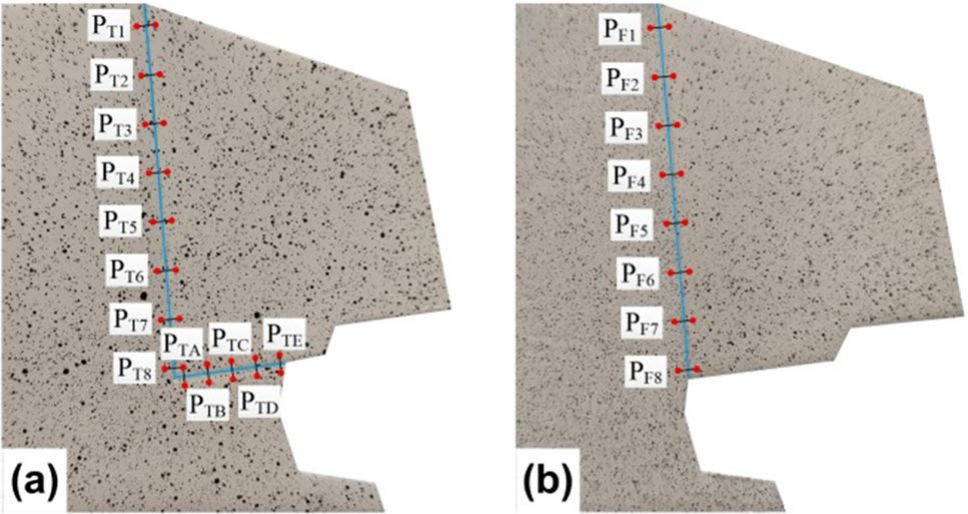
Schematic diagram of monitoring point selection.

To explore the failure behavior of dangerous rocks from a quantitative perspective, the evolution characteristics of the relative displacement along the joint plane and sand-mudstone interface were investigated in this section. Based on the DIC calculation, the relative displacements (including tangential and normal) along the joint and bedded planes can be extracted from the full-field displacement field. First, the displacement field of the model surface in the global coordinate system is obtained. Then, the coordinate conversion rules are developed according to the distribution of the joint plane or sand-mudstone interface. Based on the above rules, the displacements in the global coordinate system are transformed into tangential and normal components parallel and perpendicular to the jointed plane (or sand-mudstone interface). Eventually, the relative normal and tangential displacements along the jointed plane (or sand-mudstone interface) can be obtained. It is specified that the normal relative displacement is positive in the open direction and the tangential relative displacement is positive in the downward direction. For toppling type dangerous rock, eight groups of monitoring points (named P_T1_, P_T2_, …, P_T8_) along the joint plane and five groups (named P_TA_, P_TB_, …, P_TE_) of monitoring points along the bedded plane are selected, as shown in Fig. 7a. For falling type dangerous rock, eight groups of monitoring points (named P_F1_, P_F2_, …, P_F8_) along the joint plane are selected, as shown in Fig. 7b. It should be noted that the line of two monitoring points in the same group is perpendicular to the joint plane (or sand-mudstone interface), and their distance to the joint plane (or sand-mudstone interface) is equal, as shown in Fig. 7.

#### Toppling failure mode

**Figure 8:**
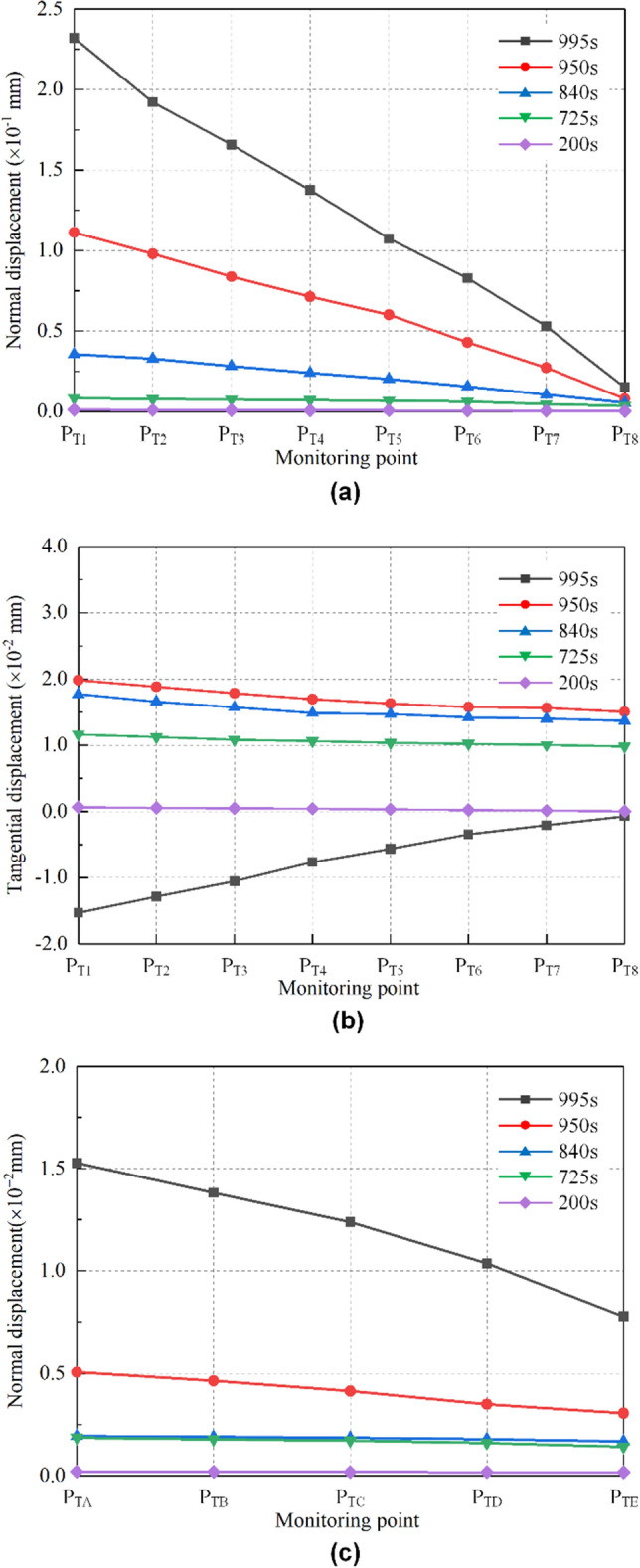
Relative displacement evolution of toppling type dangerous rock. (**a**) Normal displacement along the joint plane. (**b**) Tangential displacement along the joint plane. (**c**) Normal displacement along the sand-mudstone interface.

The relative displacement evolution of the monitoring points on the toppling type dangerous rock at typical moments is shown in Fig. 8. The normal and tangential displacements along the joint plane and the normal displacements along the sand-mudstone interface will be analyzed below to reveal the failure behavior of the toppling type dangerous rock.

Normal displacements along the joint plane are shown in Fig. 8a. When *t* = 200 s, the relative displacements are close to 0, indicating that the dangerous rock block is stable at this time. When *t* = 725 s, 840 s and 950 s, the relative displacements of each monitoring point gradually increased, indicating that the dangerous rock block gradually developed from a stable state to an unstable state. At 995 s, the dangerous rock block is about to be destabilized, at which time the relative displacements of each monitoring point continue to increase and reach the maximum values before destabilization. In addition, the relative displacements gradually decrease as the monitoring points are positioned near the bottom of the joint plane. It indicates that the separation between the dangerous rock block and the stable bedrock block gradually develops from the upper part of the joint plane to the lower part.

Tangential displacements along the joint plane are shown in Fig. 8b; similarly, the relative displacements are close to 0 at 200 s, and the dangerous rock block is stable. With increasing time (at 725 s, 840 s and 950 s), the relative displacements of all monitoring points show different degrees of positive growth. It can be explained by the thawing of the ice layer on the sand-mudstone interface causing a slight drop of the dangerous rock block in the test. Interestingly, this phenomenon can be regarded as the breaking of the structural plane filler under pressure. In addition, the relative displacements of all monitoring points are relatively close at the above four moments (*t* = 200 s, 725 s, 840 s and 950 s), indicating that the fall of the dangerous rock block is uniform. Before the destabilization failure of the dangerous rock block, that is, *t* = 995 s, the relative displacement exhibits negative growth, which indicates that the dangerous rock block is moving upward at this time. As the monitoring position approaches the bottom of the joint plane, the relative displacement gradually decreases, and the relative displacement of the bottom monitoring point is close to 0.

Normal displacements on the sand-mudstone interface are shown in Fig. 8c; similar to the variation law of normal relative displacements of the joint plane, the normal relative displacements of the sand-mudstone interface increase gradually with time. Meanwhile, the relative displacements are all positive (that is, the opening state), indicating that the upward movement displacement of the dangerous rock block is greater than the displacements from the ice layer thawing. In addition, the relative displacements gradually decrease as the monitoring points are positioned to the right of the sand-mudstone interface. This indicates that the separation between the dangerous rock block and the stable bedrock block gradually develops from the left part of the sand-mudstone interface to the right part.

#### Falling failure mode

**Figure 9:**
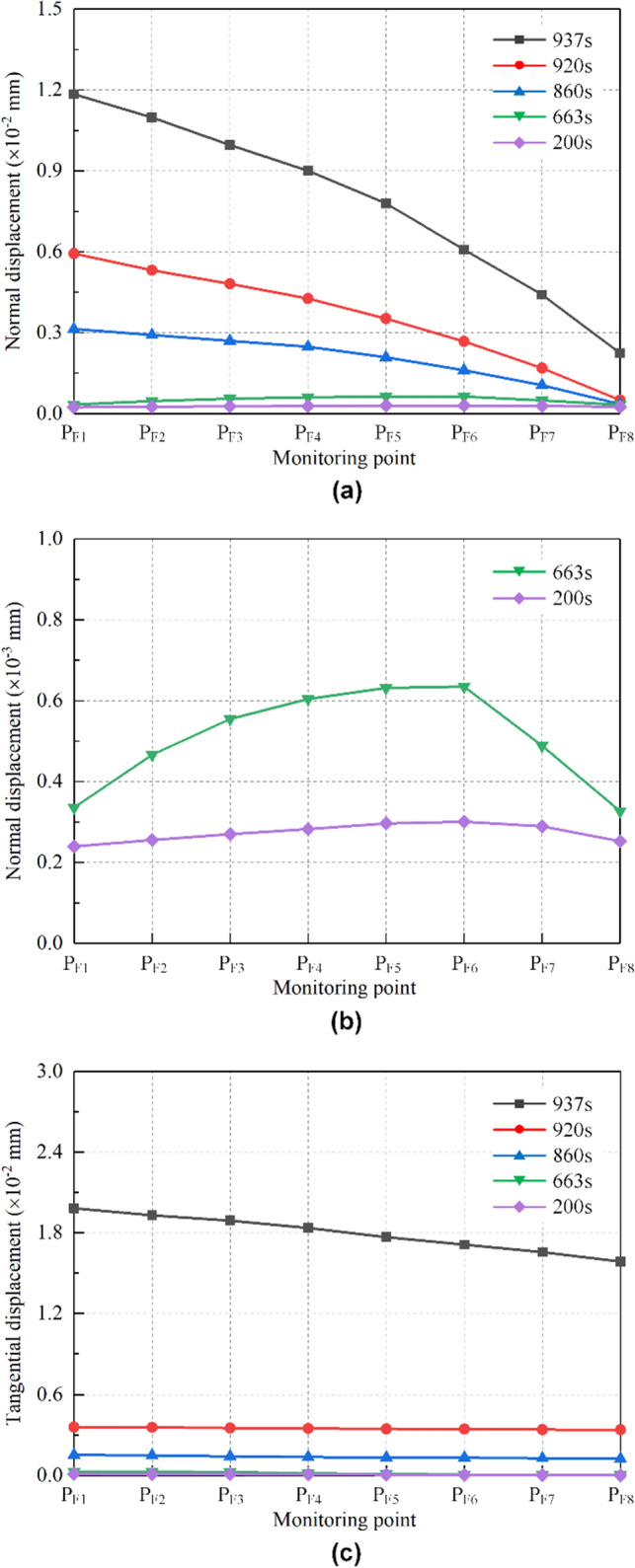
Relative displacement evolution along the joint plane of falling type dangerous rock. (**a**) Normal displacement along the joint plane. (**b**) Normal displacement along the joint plane based on local amplification. (**c**) Tangential displacement along the joint plane.

The relative displacement evolution of the monitoring points on the falling type dangerous rock at typical moments is shown in Fig. 9. The normal and tangential displacements along the joint plane are analyzed below to reveal the failure behavior of the falling type dangerous rock.

Normal displacements along the joint plane are shown in Fig. 9a, and the relative normal displacement evolution of falling type dangerous rock is almost similar to that of toppling type dangerous rock. However, at 200 s and 663 s, the separation of the joint plane develops from the lower middle part toward the two ends, which can be observed more clearly in Fig. 9b. When *t* = 725 s, 840 s and 950 s, the separation between the dangerous rock block and stable bedrock block develops from top to bottom along the joint plane. Moreover, the relative displacement values of falling type dangerous rock are very small compared with toppling type one. In particular, it is noted that the falling type dangerous rock is observed to be horizontally separated from the stable bedrock block because the joint plane is inclined.

Tangential displacements along the joint plane are shown in Fig. 9c. At 200 s and 663 s, the relative displacements of all monitoring points are close to 0, indicating that the dangerous rock block is not moving along the joint plane. At 860 s and 920 s, due to the weakening of the ice layer adhesive force and the overhang of the dangerous rock block, the dangerous rock block undergoes a small downward slip along the joint plane. The slip distances of these monitoring points are the same. When *t* = 937 s, the relative displacement of each monitoring point shows a significant increase because the dangerous rock block is about to produce destabilization failure. Likewise, as the monitoring position approaches the bottom of the joint plane, the relative displacement gradually decreases.

## Failure mechanism of the dangerous rocks

### The viewpoint from relative displacement

**Figure 10:**
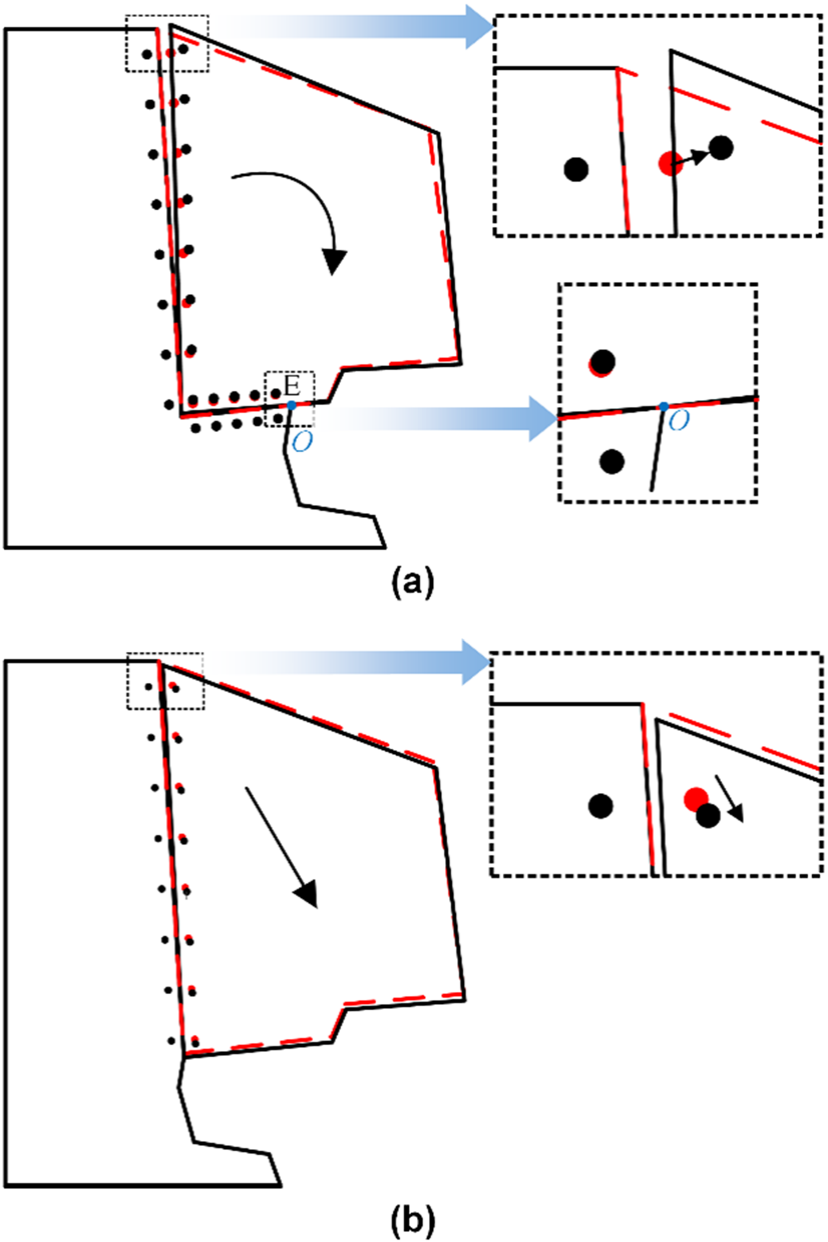
Failure mechanism of the dangerous rock based on local deformation. (**a**) The toppling failure mode. (**b**) The falling failure mode.

The failure mechanisms of the two types of dangerous rocks are summarized by the displacement evolution analysis described above, as shown in Fig. 10. The dangerous rock block before and after deformation is indicated by the red dashed box and black solid box, respectively. The monitoring points before and after deformation are indicated by the red dots and black dots, respectively.

The motion trends of monitoring points P_T1_ and P_TE_ can be found through the locally enlarged diagram, as shown in Fig. 10a. Combined with the aforementioned displacement analysis, the failure mechanism of the toppling type dangerous rock can be obtained. The failure of the toppling dangerous rock is dominated by tensile failure. The separation between the dangerous rock block and the bedrock gradually extends downward from the top and eventually rotates around fulcrum O, leading to destabilization failure, as shown in Fig. 10a.

The motion trend of monitoring point P_F1_ can be found through the enlarged local diagram, as shown in Fig. 10b. Combined with the aforementioned displacement analysis, the failure mechanism of the falling type dangerous rock can be obtained. The failure of the falling type dangerous rock is dominated by tensile‒shear failure. Specifically, the strength of the joint plane starts to weaken from the top, and the dangerous rock block slides down slowly along the joint plane as a whole. Eventually, when the adhesive force of the joint plane is not enough to ensure the stability of the dangerous rock block, it falls rapidly to form destabilization failure, as shown in Fig. 10b.

### The viewpoint from the displacement vector

The surface displacement fields of the model specimens are calculated by Ncorr software in this study, and the horizontal and vertical displacement field data matrices are extracted. Then, the vector solution is performed in the MATLAB environment by using Eq. (1) to obtain the displacement vector of each model specimen. The coordinates of any calculated point on the reference digital image are assumed to be (*x*, *y*), and the deformed coordinates are (*x*′, *y*′); then, the solution of the displacement vector can be achieved according to the following function:

1 $$\left\{ \begin{gathered} l = \sqrt {\left( {x - x^{\prime}} \right)^{2} + \left( {y - y^{\prime}} \right)^{2} } \hfill \\ \alpha = \arctan \frac{\Delta y}{{\Delta x}} = \arctan \frac{{\left( {y - y^{\prime}} \right)}}{{\left( {x - x^{\prime}} \right)}} \hfill \\ \end{gathered} \right.,$$

where *l* is the total displacement of the geometric point and *α* is the angle between the direction of the displacement vector and the positive direction of the *x*-axis, specified horizontal to the right as the *x*-positive direction.

In this study, the full-field displacement vectors before destabilization of the toppling and falling dangerous rocks are calculated to reveal their failure mechanism from the field perspective.

**Figure 11:**
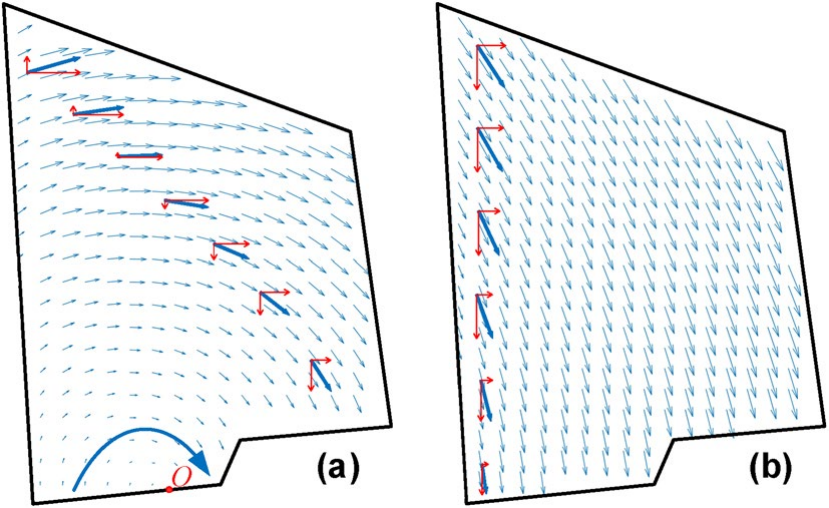
The full-field displacement vectors before destabilization. (**a**) The toppling failure mode; (**b**) The falling failure mode.

1. Toppling failure mode: As shown in Fig. 11a, it is observed that the displacement vectors on the surface of the dangerous rock block show an overall clockwise rotational motion, and the horizontal components of their displacement vectors are all oriented to the right, which indicates that the perilous rock has a tendency to open to the right. The transformation in the direction of the vertical displacement component of the fine-viewing particles is easily detected to be upward with the left side at point O and downward to the right of point O. As a result, it is proven that the toppling dangerous rocks rotate around the intersection between the laminated plane and the free surface. Therefore, the toppling dangerous rocks are mainly destabilized due to tensile failure. It is worth mentioning that the displacement vectors of the dangerous rock block calculated by the DIC method are similar to those calculated by Zhang 5] by simulation, which further illustrates the reliability of the results.
2. Falling failure mode: As shown in Fig. 11b, the fine particles on the dangerous rock block specimen surface show an overall shear motion toward the right and downward direction. Meanwhile, the displacement vector inclination is increasingly close to the joint plane inclination from top to bottom, which indicates the top-down progressive cracking behavior along the joint plane. Therefore, the falling dangerous rocks are mainly destabilized due to tensile‒shear failure.

## Failure precursor identification and early warning of dangerous rocks

**Figure 12:**
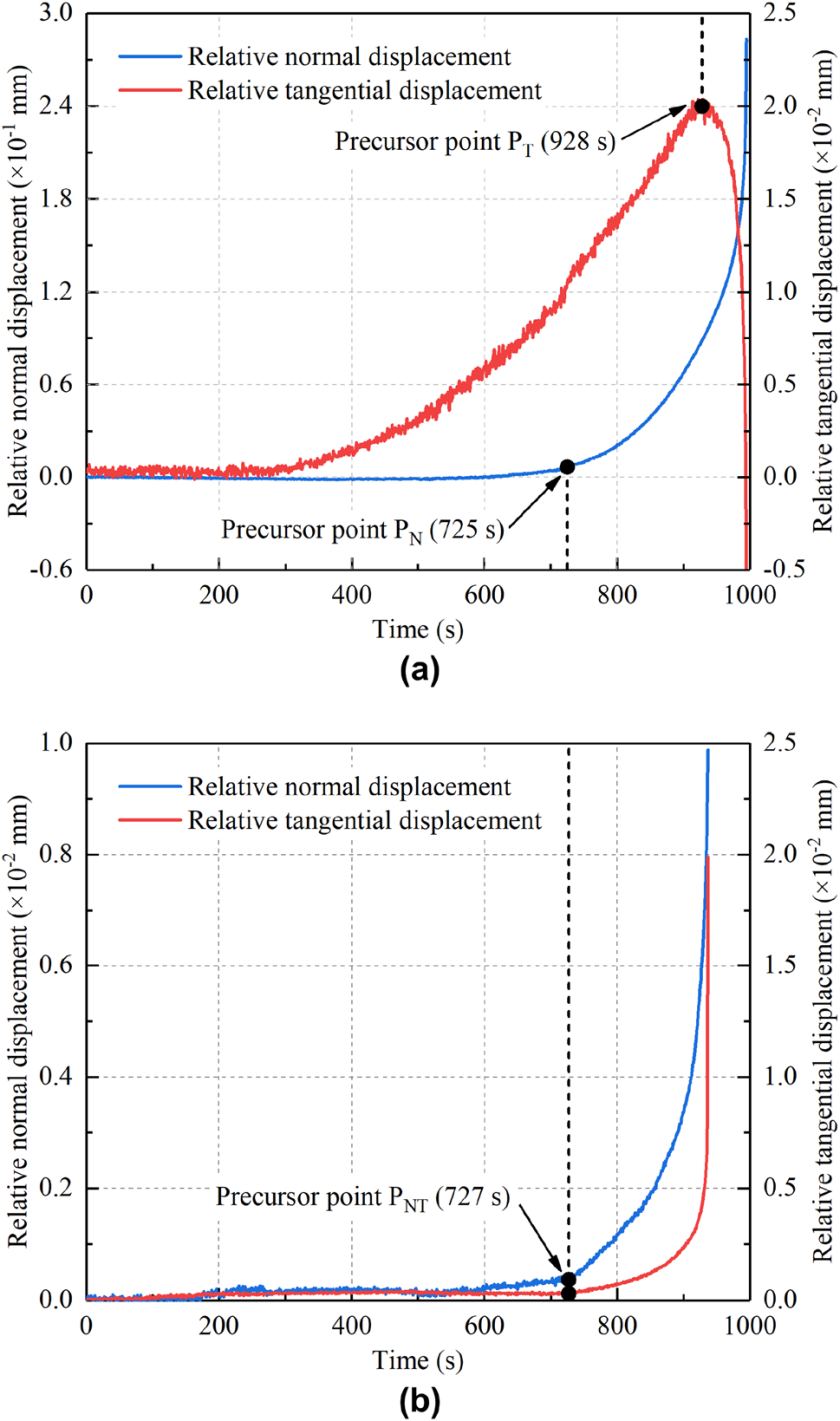
Relative displacement‒time curves of the key monitoring points. (**a**) The toppling failure mode. (**b**) The falling failure mode.

From Figs. 8 and 9, it can be seen that monitoring points P_T1_ in the toppling type dangerous rock and P_F1_ in the falling dangerous rock show the most significant spatial and temporal distribution characteristics. Therefore, the evolution curves for the relative displacement of the abovementioned two monitoring points with time are obtained, as shown in Fig. 12.

Obvious anomalous features are observed in the relative displacement evolution curves. These anomalies are related to the local movement or deformation of the dangerous rock block, which can be used as precursor points for the destabilization failure of the dangerous rock. For the toppling type dangerous rock specimen, the turning point of the relative normal displacement from almost zero to the acceleration beginning can be recognized as a precursor point (P_N_), as shown in Fig. 12a. The appearance of the precursor point P_N_ is related to the fracture at the top of the potential joint plane, at which the dangerous rock block has a tendency to separate from the stable bedrock block. A peak is observed in the evolution curve of the relative tangential displacement, which can be regarded as a precursor point (P_T_), as shown in Fig. 12a. The appearance of the precursor point P_T_ indicates a tendency for the dangerous rock block to rotate upward around fulcrum O. For the falling-type dangerous rock specimen, the turning point of the relative normal and relative tangential displacements from almost zero to the beginning of acceleration can be recognized as a precursor point (P_NT_), as shown in Fig. 12b. The appearance of precursor point P_NT_ is due to the weakening of the joint plane adhesive force, which in turn makes there is a tendency for the dangerous rock block to separate from the stable bedrock block and fall downward.

Table 1 summarizes the precursor points based on the evolution characteristics of the relative displacement. The time ratio (TR) is defined as the appearance time of the precursor point divided by the dangerous rock failure time. The early warning interval length (EWIL), which is defined as the dangerous rock failure time reduced by the appearance time of the precursor point. The TR and EWIL provide quantitative information on how close the appearance of precursor points is to the destabilization failure time of the dangerous rock. Therefore, a positive effect for early warning of dangerous rock can be provided by the precursor identification method based on the relative displacement evolution of the key points. In addition, the precursor information of the toppling type dangerous rock is more sensitive than that of the falling type dangerous rock, which is closely related to its failure mode. Additionally, it is more difficult to obtain an early warning for falling type dangerous rock. Therefore, more attention should be given to safety prevention and stability assessment in the project.

**Table 1:** Summary of the precursor points based on the evolution characteristics of relative displacement.

Failure mode	Precursor point	Precursor time (s)	Failure time (s)	TR (%)	EWIL (s)
Toppling	P_N_	725	996	72.79	271
	P_T_	928	996	93.17	68
Falling	P_NT_	727	938	77.51	211

It should be noted that all the findings in this paper are obtained by laboratorial frozen-thawing test. Actually, the failure precursor of dangerous rock is influenced by many factors, such as lithology, fracture characteristics, geometry and geotechnical parameters^27^. Therefore, the relevant conclusions need to be further corroborated by a large number of indoor experiments. In addition, the size effects of the precursor need to be further investigated when the small-size test results of this study are applied to hazard warning of dangerous rock projects. Therefore, the establishment of engineering-scale precursor features is also the focus and challenge of future study.

## Conclusions

In the present study, two conceptual models of dangerous rocks in a typical rock fall-prone area are established. The stereolithography apparatus 3DP technology is utilized to prepare the models of toppling type and falling type dangerous rocks. The failure behavior and mechanical mechanism of dangerous rocks are studied through laboratorial frozen-thawing tests and DIC. The main conclusions of this paper are as follows:

1. The 3D printed models are capable of accurately duplicating the geometry of the dangerous rock. The frozen-thawing test (FTT) is conducted to reproduce the failure process of the toppling mode and falling mode dangerous rocks.
2. The DIC-based strain fields at five typical moments show that the strain localization phenomenon can reflect the failure state of dangerous rocks. The failure behaviors of dangerous rocks are characterized by calculating the relative normal and tangential displacements along the jointed plane (or sand-mudstone interface) at the above five typical moments. Before the destabilization of toppling type dangerous rock, the separation of the joint plane gradually extends downward from the top. While for falling type dangerous rock, the separation of the joint plane initially develops from the lower middle part toward the two ends and then extends downward from the top.
3. Two viewpoints are employed to reveal the failure mechanisms of unstable rocks. Based on the motion trend of the monitoring points, a rotational failure mechanism is observed for the toppling type dangerous rock, and the falling type one is dominated by downward tensile‒shear failure. The same failure mechanisms are found from the displacement vector fields of two dangerous rock blocks.
4. The relative displacement evolution curves of monitoring points P_T1_ and P_F1_ show obvious abnormal features during the test. Therefore, a DIC-based early warning method is proposed by identifying precursor points, wherein TR and EWIL can provide a positive warning effect for the ultimate instability of dangerous rocks.

## Data Availability

Data analysed in this study are available upon reasonable request from the corresponding author (Kai Zhang).

## References

[1] Ma K, Liu GY (2021). Three-dimensional discontinuous deformation analysis of failure mechanisms and movement characteristics of slope rockfalls. Rock Mech. Rock Eng..

[2] Yan JH, Chen JP, Zhou FJ, Zhang W, Zhang YS, Zhao MY, Ji YP, Liu YQ, Xu WL, Wang Q (2022). A new framework for geometrical investigation and stability analysis of high-position concealed dangerous rock blocks. Acta Geotech..

[3] Yu B, Yi W, Zhao HB (2017). Experimental study on the maximum impact force by rock fall. Landslides.

[4] Hungr O, Leroueil S, Picarelli L (2013). The varnes classification of landslide types, an update. Landslides.

[5] Zhang K, Tan P, Ma GW, Cao P (2015). Modeling of the progressive failure of an overhang slope subject to differential weathering in three gorges reservoir, China. Landslides.

[6] Wu LZ, Li B, Huang RQ, Wang QZ (2016). Study on mode I–II hybrid fracture criteria for the stability analysis of sliding overhanging rock. Eng. Geol..

[7] Huang RQ, Wu LZ, He Q, Li JH (2017). Stress intensity factor analysis and the stability of overhanging rock. Rock Mech. Rock Eng..

[8] Spreafico MC, Cervi F, Francioni M, Stead D, Borgatti L (2017). An investigation into the development of toppling at the edge of fractured rock plateaux using a numerical modelling approach. Geomorphology.

[9] Bolla A, Paronuzzi P (2019). Numerical investigation of the pre-collapse behavior and internal damage of an unstable rock slope. Rock Mech. Rock Eng..

[10] Shi C, Yang B, Zhang YP, Yang JX (2020). Application of discrete-element numerical simulation for calculating the stability of dangerous rock mass: A case study. Int. J. Geomech..

[11] Wu Y, Li XP, Zhu L (2021). Fracture mechanism of rock collapse in the freeze–thaw zone of the eastern sichuan–tibet mountains under seasonal fluctuating combinations of water and heat. Nat. Hazards.

[12] Wang LQ, Yin YP, Zhou CY, Huang BL, Wang WP (2020). Damage evolution of hydraulically coupled jianchuandong dangerous rock mass. Landslides.

[13] Yin YP, Wang LQ, Zhang WG, Zhang ZH, Dai ZW (2022). Research on the collapse process of a thick-layer dangerous rock on the reservoir bank. Bull. Eng. Geol. Environ..

[14] Zhu JB, Zhou T, Liao ZY, Sun L, Li XB, Chen R (2018). Replication of internal defects and investigation of mechanical and fracture behaviour of rock using 3d printing and 3d numerical methods in combination with X-ray computerized tomography. Int. J. Rock Mech. Min. Sci..

[15] Park S, Shin C, Kim Y, Clayton RW (2022). Seismic wave simulation using a 3d printed model of the Los Angeles Basin. Sci. Rep..

[16] Que XC, Zhu ZD, He YX, Niu ZH, Huang HN (2022). Strength and deformation characteristics of irregular columnar jointed rock mass: A combined experimental and theoretical study. J. Rock Mech. Geotech. Eng..

[17] Song LB, Jiang Q, Zhong Z, Dai F, Wang G, Wang XK, Han GS, Zhang D (2022). Technical path of model reconstruction and shear wear analysis for natural joint based on 3d scanning technology. Measurement.

[18] Wang YY, Wang L, Zhang WG, Ma GW (2022). Size effect of fractured rock mass based on 3d printed model testing. Rock Mech. Rock Eng..

[19] Sharafisafa M, Shen L, Xu Q (2018). Characterisation of mechanical behaviour of 3d printed rock-like material with digital image correlation. Int. J. Rock Mech. Min. Sci..

[20] Sharafisafa M, Aliabadian Z, Tahmasebinia F, Shen L (2021). A comparative study on the crack development in rock-like specimens containing unfilled and filled flaws. Eng. Fract. Mech..

[21] Aliabadian Z, Zhao GF, Russell AR (2019). Failure, crack initiation and the tensile strength of transversely isotropic rock using the Brazilian test. Int. J. Rock Mech. Min. Sci..

[22] Yang HT, Lin H, Wang YX, Cao RH, Li JT, Zhao YL (2021). Investigation of the correlation between crack propagation process and the peak strength for the specimen containing a single pre-existing flaw made of rock-like material. Arch. Civ. Mech. Eng..

[23] Ma WB, Chen YL, Yi W, Guo SC (2022). Investigation on crack evolution behaviors and mechanism on rock-like specimen with two circular-holes under compression. Theor. Appl. Fract. Mech..

[24] Tan LH, Zhou ZL, Cai X, Rui YC (2022). Analysis of mechanical behaviour and fracture interaction of multi-hole rock mass with DIC measurement. Measurement.

[25] Luo P, Wang L, Li D, Yang J, Lv X (2022). Deformation and failure mechanism of horizontal soft and hard interlayered rock under uniaxial compression based on digital image correlation method. Eng. Fail. Anal..

[26] Miščević P, Vlastelica G (2014). Impact of weathering on slope stability in soft rock mass. J. Rock Mech. Geotech. Eng..

[27] Cano M, Tomás R (2013). Characterization of the instability mechanisms affecting slopes on carbonatic flysch: Alicante (se Spain), case study. Eng. Geol..

[28] Local Standard of Chongqing City (2004) Design specifications of implementation project for geologic hazards (DB50/5029–2004). (in Chinese)

[29] Zhou T, Zhu JB, Xie HP (2020). Mechanical and volumetric fracturing behaviour of three-dimensional printing rock-like samples under dynamic loading. Rock Mech. Rock Eng..

[30] Du Y, Xie MW, Jiang YJ, Li B, Chicas S (2016). Experimental rock stability assessment using the frozen–thawing test. Rock Mech. Rock Eng..

[31] Du Y, Lu YD, Xie MW, Jia JL (2020). A new attempt for early warning of unstable rocks based on vibration parameters. Bull. Eng. Geol. Environ..

[32] Huo LC, Du Y, Xie MW, Zhang XY, Jia BN, Cong XM (2021). Unstable rock mass identification method based on time and frequency domain dynamic parameters. Chin. J. Rock Mech. Eng..

